# Etoposide damages female germ cells in the developing ovary

**DOI:** 10.1186/s12885-016-2505-9

**Published:** 2016-08-11

**Authors:** Agnes Stefansdottir, Zoe C. Johnston, Nicola Powles-Glover, Richard A. Anderson, Ian R. Adams, Norah Spears

**Affiliations:** 1 Centre for Integrative Physiology, University of Edinburgh, Edinburgh, EH8 9XD UK; 2 AstraZeneca, Alderley Park, Macclesfield, SK10 4TG UK; 3 MRC Centre for Reproductive Health, University of Edinburgh, Edinburgh, EH16 4TJ UK; 4 MRC Human Genetics Unit, MRC Institute of Genetics and Molecular Medicine, University of Edinburgh, Edinburgh, EH4 2XU UK; 5 Present Address: Institute of Biodiversity, Animal Health and Comparative Medicine, University of Glasgow, Glasgow, G61 1QH UK

**Keywords:** Etoposide, Oogonia, Oocyte, Ovarian follicle, Chemotherapy, Tissue culture, Fetal ovary

## Abstract

**Background:**

As with many anti-cancer drugs, the topoisomerase II inhibitor etoposide is considered safe for administration to women in the second and third trimesters of pregnancy, but assessment of effects on the developing fetus have been limited. The purpose of this research was to examine the effect of etoposide on germ cells in the developing ovary. Mouse ovary tissue culture was used as the experimental model, thus allowing us to examine effects of etoposide on all stages of germ cell development in the same way, in vitro.

**Results:**

Fetal ovaries from embryonic day 13.5 CD1 mice or neonatal ovaries from postnatal day 0 CD1 mice were cultured with 50–150 ng ml^−1^ or 50–200 ng ml^−1^ etoposide respectively, concentrations that are low relative to that in patient serum. When fetal ovaries were treated prior to follicle formation, etoposide resulted in dose-dependent damage, with 150 ng ml^−1^ inducing a near-complete absence of healthy follicles. In contrast, treatment of neonatal ovaries, after follicle formation, had no effect on follicle numbers and only a minor effect on follicle health, even at 200 ng ml^−1^. The sensitivity of female germ cells to etoposide coincided with topoisomerase IIα expression: in the developing ovary of both mouse and human, topoisomerase IIα was expressed in germ cells only prior to follicle formation.

**Conclusions:**

Exposure of pre-follicular ovaries, in which topoisomerase IIα expression was germ cell-specific, resulted in a near-complete elimination of germ cells prior to follicle formation, with the remaining germ cells going on to form unhealthy follicles by the end of culture. In contrast, exposure to follicle-enclosed oocytes, which no longer expressed topoisomerase IIα in the germ cells, had no effect on total follicle numbers or health, the only effect seen specific to transitional follicles. Results indicate the potential for adverse effects on fetal ovarian development if etoposide is administered to pregnant women when germ cells are not yet enclosed within ovarian follicles, a process that starts at approximately 17 weeks gestation and is only complete towards the end of pregnancy.

**Electronic supplementary material:**

The online version of this article (doi:10.1186/s12885-016-2505-9) contains supplementary material, which is available to authorized users.

## Background

Cancer is diagnosed in approximately one out of every 1000 pregnancies, often requiring consideration of chemotherapy administration to the pregnant woman [1–4]. Chemotherapy administration during the first trimester is now largely avoided, as it is associated with increased risk of congenital malformations and high risk of spontaneous abortion [5]. However, it is now widely considered that chemotherapy treatment during the second and third trimesters of pregnancy is relatively safe for the developing fetus (for example, [6–8]), with several studies showing no congenital malformations in infants born to women receiving chemotherapy at that time [1, 3, 5, 6]. Associations have however been made between chemotherapy treatment during pregnancy and increased risk of intrauterine growth restriction, miscarriage, preeclampsia and stillbirth [4, 9].

Effects of chemotherapy agents on ovarian function and fertility in girls and young women treated for cancer are well recognised and may result in infertility and premature ovarian insufficiency (POI) [10, 11]. In contrast, there is a distinct lack of information on the long-term effects of chemotherapy treatment on the future fertility of female fetuses when exposure occurs during the particularly vulnerable window of female germ cell development during fetal life [12], although cyclophosphamide exposure *in utero* results in markedly reduced follicle numbers in neonatal mice [13]. Drug exposure to the developing ovary could have toxic effects on germ and/or ovarian somatic cells, with the consequences of such effects unlikely to manifest themselves until at least after puberty. Additionally, any genetic damage to germ cells during fetal development could then be passed on to subsequent generations, the ‘grand-maternal’ effect [14].

**Fig. 1:**
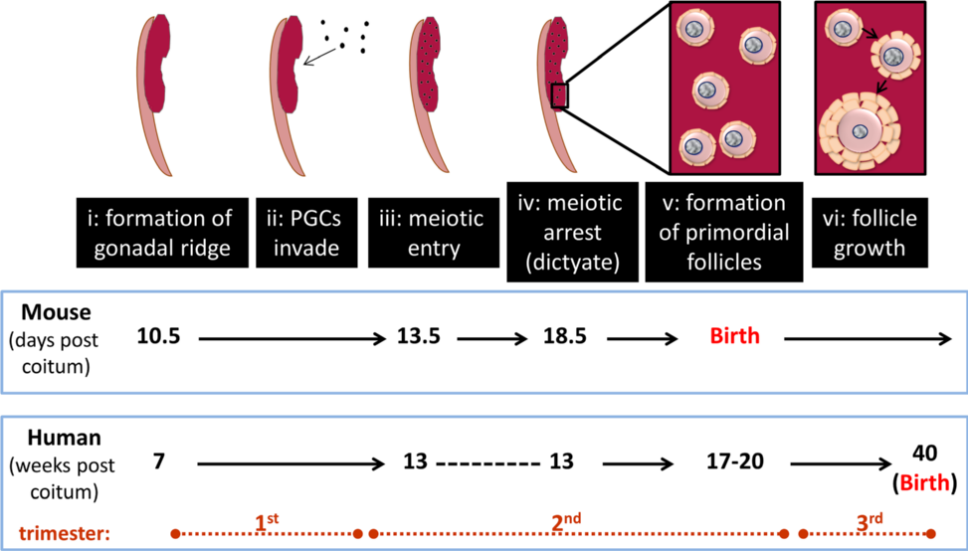
Timing of ovary development in mouse and human. The gonadal ridge forms during fetal development (*i*), after which it is invaded by proliferating primordial germ cells (*ii*). Meiosis is then initiated, prior to ovarian follicle formation (*iii*). After entering Prophase I of meiosis, germ cells, now termed oocytes, become arrested at the dictyate stage of prophase I (*iv*), at which point they interact with surrounding somatic pregranulosa cells to form primordial follicles (*v*). From this point on, follicles gradually leave the resting primordial phase and initiate growth (*vi*)

Formation of the gonadal ridge begins around week 7 of human gestation, embryonic day 10.5 (E10.5) in the mouse fetus. Shortly after this, proliferating primordial germ cells invade the developing ovary. After a further short proliferative phase, the germ cells initiate meiosis, which subsequently arrests at the diplotene stage of prophase I. Around that time, the germ cells, now termed oocytes, interact with surrounding somatic pregranulosa cells to form primordial follicles (PFs): meiotic arrest and follicle formation start between weeks 17 to 20 of human gestation, although follicle formation can continue until late in pregnancy [15]; in the mouse, oocytes have entered meiotic arrest by the end of gestation, with follicle formation occurring around the time of birth. PF formation, therefore, takes place throughout the second and into the third trimester of human fetal development, compared with peri-natal formation in the mouse (Fig. 1). Once PFs have formed, there is continual ‘release’ of PFs from that resting follicle pool, follicles then undergoing growth initiation, with the potential to develop through to the preovulatory stage. The number of PFs formed during fetal life is crucial for future fertility, with the size of the PF pool directly related to a female’s reproductive lifespan [16].

Etoposide is commonly used in the treatment of ovarian and lung cancers, leukemias and lymphomas. It may be administered during pregnancy [1, 17, 18], and has been considered safe for the fetus if given during the second or third trimester, with births of healthy babies reported (e.g. [19–21]. However, no data have been reported on possible detrimental effects of etoposide on the reproductive systems of these children, with none having yet reached puberty. Etoposide acts by inhibiting the enzyme type II Topoisomerase (Topo II). Topo II catalyses topological transitions in double-stranded DNA, and thus influences transcription, DNA replication, chromosome condensation and the separation of sister chromatids during mitosis. It creates transient double strand breaks in DNA, relieving the torsional stress created when DNA strands become supercoiled and allowing the passage of the intact DNA segment through the cleaved strand, after which it re-seals the double strand break [22–24]. Etoposide acts by interfering with the ability of Topo II to re-ligate the nick in the DNA strand, consequently increasing DNA fragmentation and inducing tumour cell death [25–28]. Mammals express two functionally distinct Topo II paralogues: Topo IIα and Topo IIβ. Topo IIα has a widespread role in resolving replication-induced DNA catenanes in proliferating cells, whereas Topo IIβ has a more restricted role, at least in neuronal development, and cannot efficiently provide the essential function of Topo IIα in proliferating cells [reviewed in 28]. In the mouse ovary, Topo IIβ is expressed in oocytes at all developmental stages, with a low expression in granulosa cells of PFs and a more pronounced expression in the granulosa cells of growing follicles [29]. Mice with Topo IIβ conditionally deleted from their granulosa cells, contain increased cell DNA damage within the granulosa cells, leading to increased follicle atresia [29]. In male germ cells, Topo II is required during prophase I of meiosis [24, 30, 31], with high levels of chromosomal aberrations present in spermatocytes treated with therapeutic doses of etoposide [32]. These spermatocytes had increased levels of acentric fragments and deletions that are associated with embryonic lethality [32]. In the female, Topo II is required for chromosome separation during oocyte meiotic maturation, but is dispensable for resumption of meiosis [31]. However, its role in early stages of female meiosis, prior to meiotic arrest, remains unclear, as does any effect of etoposide. If a similar effect on meiosis occurring in female germ cells to that which occurs in the male would affect human ovarian development during the second and third trimesters of pregnancy.

We report here on the effects of etoposide on female germ cell development. In the mouse, our experimental model, follicle formation occurs around the time of birth, thus requiring manipulation of both fetal and neonatal ovaries. An initial objective of the work described here was the development of a mouse fetal ovary culture system that traverses both fetal and neonatal stages of germ cell development, thus allowing us to examine effects of etoposide on all stages of germ cell development in a consistent manner, in vitro. This novel fetal ovary culture technique supports survival of pre-meiotic germ cells, progression through prophase I of meiosis up to meiotic arrest, followed by follicle formation and subsequent growth initiation, and has been used here alongside an established neonatal ovary culture system in which oocytes have already entered meiotic arrest prior to culture [33, 34]. In vitro models are a growing area in reproductive toxicology research, allowing pragmatic and mechanistic studies of action of chemicals. The rodent ovary is an excellent model for in vitro studies, partly due to its small size, short time-course, high material availability, and similarities in many key developmental and functional aspects with the human ovary. A second aim was to determine the expression pattern of mammalian Topo IIα in the ovary, which has not yet been reported: it has been examined here, in mouse and human. The main goal of this work was to use ovarian tissue culture methods to investigate the effect of etoposide on germ cells prior to and following follicle formation. Our results show that germ cells are particularly sensitive to etoposide damage prior to follicle formation, the only stage at which both Topo IIα and TopoIIβ are expressed in the oocyte in the developing mouse and human ovary.

## Results

### Tissue culture supports physiological development of fetal mouse ovaries

**Fig. 2:**
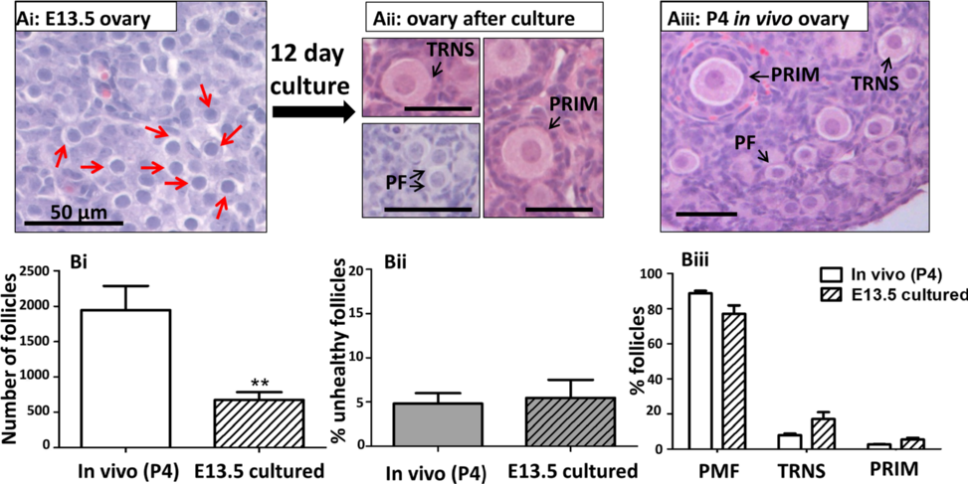
Fetal ovary culture supports follicle formation and further development to the primary stage, as occurs in vivo. Representative histological sections of cultured E13.5 mouse ovary prior to (*Ai*), and after (*Aii*) 12 days of culture, compared with in vivo P4 mouse ovary (*Aiii*). At the end of culture, healthy primordial, transitional and primary follicles formed within cultured ovaries (*Aii*), morphologically highly similar to those found in uncultured ovaries of equivalently aged mice (P4) (*Aiii*). Despite a smaller number of follicles in cultured ovaries (*Bi*), very few follicles were unhealthy, with no difference compared to the percentage of unhealthy follicles found in in vivo ovaries (*Bii*), and with follicles present at developmental stages in ratios comparable to those present in P4 in vivo ovaries (*Biii*). Scale bars: 50 μm. Histogram bars denote mean ± SEM; *n* = 5 for all groups. Stars denote significant differences relative to control (**p < 0.01)

**Fig. 3:**
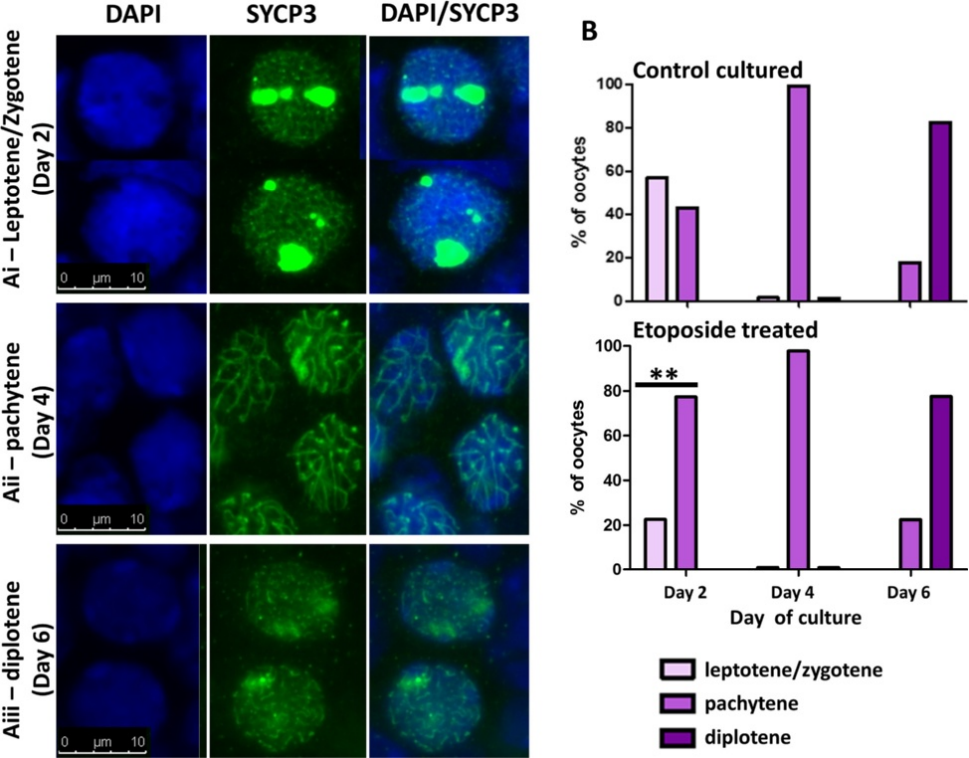
Oocytes from cultured fetal ovaries progress through prophase I of meiosis in the presence or absence of etoposide. Fetal ovaries cultured for 2, 4 or 6 days were stained for Sycp3 in order to assess the synaptonemal complex (SC). Oocytes were categorised into leptotene/zygotene, (SC assembling but not fully formed) by the presence of extensive networks of fine Sycp3 threads, often with large nuclear aggregates of Sycp3 that has not yet assembled into SC (*Ai*); pachytene, (fully synapsed SC) by the presence of a thicker well-spaced long Sycp3 threads (*Aii*); or diplotene, (SC disassembling but still present) by the presence of short thick fragments of Sycp3 threads (*Aiii*). B: Meiotic progression was examined in germ cells from control ovaries and from ovaries exposed to 150 ng ml^−1^ etoposide during culture. Control oocytes progressed through the early stages of prophase I in a normal manner during the first 6 days of culture, with the majority at leptotene/zygotene at Day 2 of culture, pachytene at Day 4 and diplotene by Day 6 of culture, immediately prior to follicle formation. Oocytes exposed to 150 ng ml^−1^ etoposide during culture were able to progress through to diplotene. At Day 2 of culture only, oocytes from etoposide-treated ovaries were at more advanced meiotic stages than those from control ovaries, but there was no difference at Days 4 or 6. Scale bars: 10 μm, *n* = 961 control oocytes and *n* = 994 treatment oocytes. Stars denote significant difference between oocytes from etoposide-exposed ovaries (*lower panel*) relative to controls (*upper panel*; **p < 0.01)

A culture technique to support development of fetal mouse ovaries from E13.5 was developed, to allow effects of etoposide to be examined both before and after follicle formation in a consistent manner, in vitro, with neonatal mouse ovary culture already an established method. Over the 12 days of culture, germ cells progressed through prophase I of meiosis, followed by formation of PFs, with some follicles subsequently initiating follicle growth to the transitional and primary follicle stage (Fig. 2Ai,ii). Cultured follicles were morphologically healthy, similar to that observed in uncultured postnatal day 4 (P4) in vivo ovaries (Fig. 2Aiii). Although there was a significant reduction in follicle numbers within cultured ovaries compared to in vivo P4 ovaries (Fig. 2Bi; *p* < 0.01, *n* = 5, from 3 independent cultures), health and follicle developmental stage were both comparable to that found in vivo (Fig. 2Bii,iii). Progression of cultured oocytes through prophase I of meiosis to diplotene was analysed through visualisation of Sycp3. Sycp3 is a component of the axial/lateral element of the synaptonemal complex (SC) that assembles during meiotic prophase I [35, 36]. In the female mouse embryo, germ cells enter meiosis at E13.5 and progress through prophase I to diplotene over the next 6–7 days [37]. Images here show that meiosis progressed in vitro as in vivo: oocytes progressed through leptotene/zygotene, pachytene, and diplotene, as assessed by SC assembly and disassembly (Fig. 3Ai-iii). In vitro oocytes were observed in all stages of prophase I, with 57.1 % still leptotene/zygotene and 42.3 % having reached pachytene by Day 2 of culture. By Day 4 of culture, the vast majority (99.2 %) had reached pachytene, and by Day 6 of culture, the majority (82.5 %) of oocytes had progressed through to the diplotene stage of prophase I (Fig. 3B, upper panel): by Day 6 of culture, no leptene/zygotene oocytes remained, as would be expected at the equivalent E19.5 point in vivo [37]. Together, these results validate the use of this fetal ovary culture system for investigations into meiotic progression and follicle formation.

### Germ cells are more vulnerable to etoposide exposure prior to follicle formation

We first tested whether etoposide might be impairing the ability of fetal oocytes to progress through meiotic prophase. Fetal mouse ovaries were immunostained for Sycp3 after culture in control medium, or in the presence of etoposide (150 ng ml^−1^) for 2, 4 or 6 days to assess progression through early prophase I. There was a difference between etoposide-treated and control oocytes at Day 2 of culture (*p* < 0.01), but the effect was no longer observed by Days 4 or 6 of culture (Fig. 3B; *p* = 0.4 at Days 4 and 6). The effect at Day 2 could be due to an initial ‘acceleration’ in meiosis in germ cells exposed to etoposide, or because the germ cells are more sensitive to etoposide at mitotic and/or pre-leptotene stages: increased sensitivity is perhaps more likely given the reduction in follicle number after etoposide exposure (see results below). Overall, results show that female germ cells are able to progress through meiosis to the diplotene stage of prophase I in the presence of etoposide.

E13.5 fetal mouse ovaries were then cultured for twelve days either in control medium throughout (Days 0–12), or exposed to a range of etoposide doses (50, 100 or 150 ng ml^−1^) for the first six days of culture (Days 0–6), followed by a further six days in control medium (Days 6–12): Day 7 of culture, when follicles have begun to form in the cultured fetal mouse ovaries, was considered as equivalent to the day of birth in vivo. Etoposide exposure occurred, therefore, prior to follicle formation, spanning entry into meiotic prophase and progression through to diplotene stage.

**Fig. 4:**
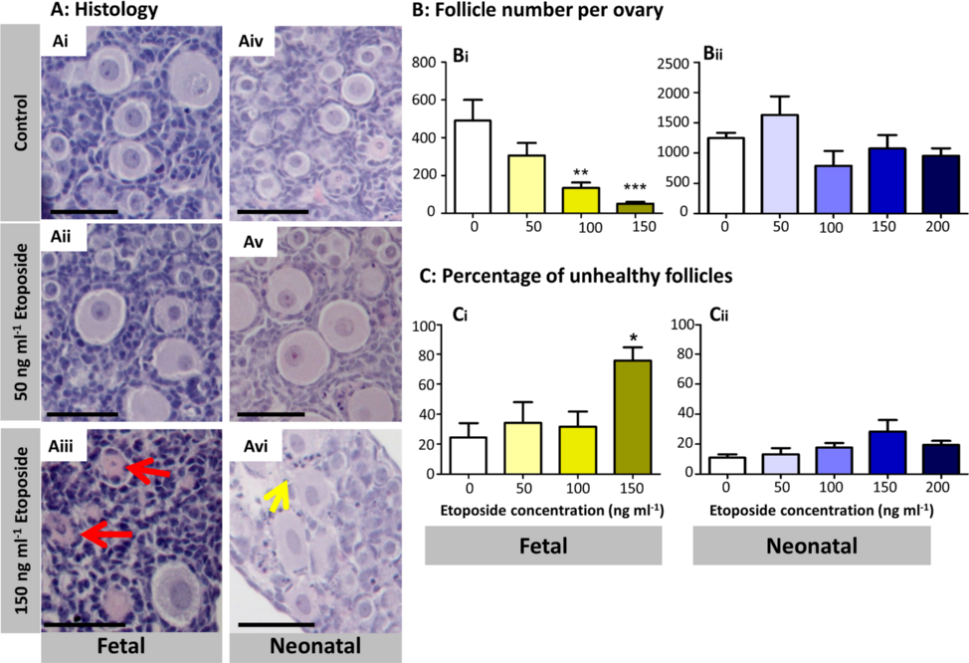
Etoposide has a significant effect on follicle numbers and health on fetal, but not neonatal ovaries. **a** Photomicrographs of haemotoxylin and eosin stained sections from fetal (*Ai-iii*) and neonatal (*Aiv-vi*) ovaries treated with etoposide in vitro. Fetal mouse ovaries cultured for twelve days in control medium (DMSO only) (*Ai*); or in the presence of 50 ng ml^−1^ (*Aii*); or 150 ng ml^−1^ (*Aiii*) etoposide for the first six days of culture. Neonatal mouse ovaries cultured for six days with control medium (DMSO only) (Aiv); or in the presence of 50 ng ml^−1^ (*Av*); or 150 ng ml^−1^ (*Avi*) etoposide for the six days of culture. **b**, **c** Follicle number (**b**) and health (**c**) was determined in cultured mouse ovaries exposed to etoposide prior to (fetal ovaries: *Bi*, *Ci*) or after (neonatal ovaries: *Bii*, *Cii*) follicle formation. Figure shows: total follicle numbers (*Bi*, *Bii*); and the percentage of follicles assessed as unhealthy (*Ci*, *Cii*). Red arrows show examples of follicles deemed unhealthy due to unhealthy oocyte; yellow arrow shows examples of follicles deemed unhealthy due to unhealthy granulosa cells. Scale bars 50 μm. Bars denote mean ± SEM; *n* = 6 for all groups. Stars denote significant differences relative to control (*p < 0.05, **p < 0.01, ***p < 0.001)

**Fig. 5:**
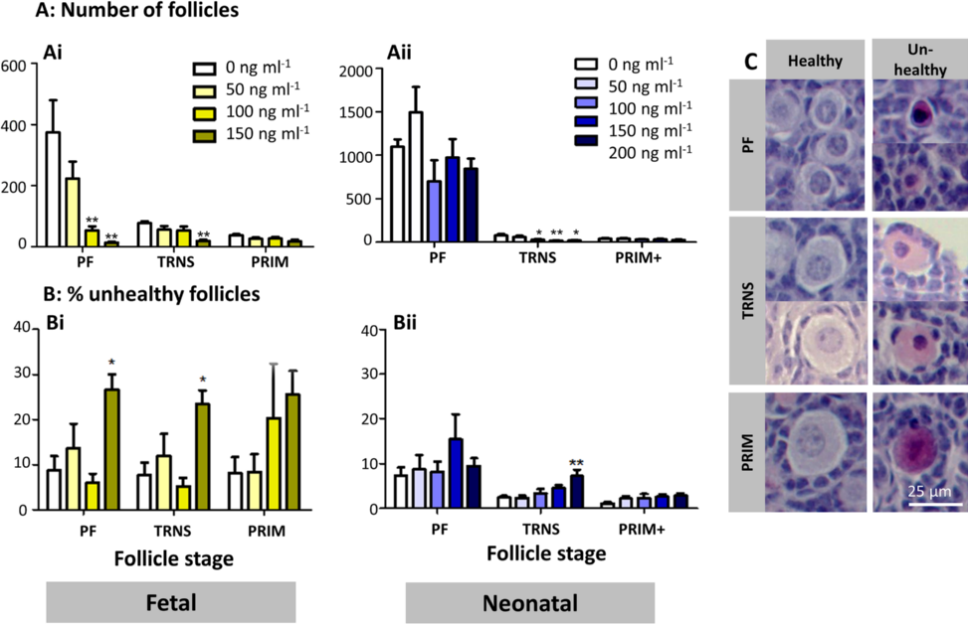
Effect of etoposide on different stages of follicle development. Number and health of primordial (PF), transitional (TRNS) and primary (PRIM) follicles was determined in cultured mouse ovaries exposed to etoposide prior to (fetal ovaries: *Ai*, *Bi*); or after (neonatal ovaries: *Aii*, *Bii*) follicle formation. Figure shows: follicle distribution (*Ai*, *Aii*); and the percentage of follicles assessed as unhealthy for each follicle stage (*Bi*, *Bii*). **c** Photomicrographs of haemotoxylin and eosin stained cultured fetal and neonatal ovary sections, comparing healthy and unhealthy primordial, transitional and primary follicles. Bars denote mean ± SEM; *n* = 6 for all groups. Stars denote significant differences relative to control (*p < 0.05, **p < 0.01, ***p < 0.001)

**Fig. 6:**
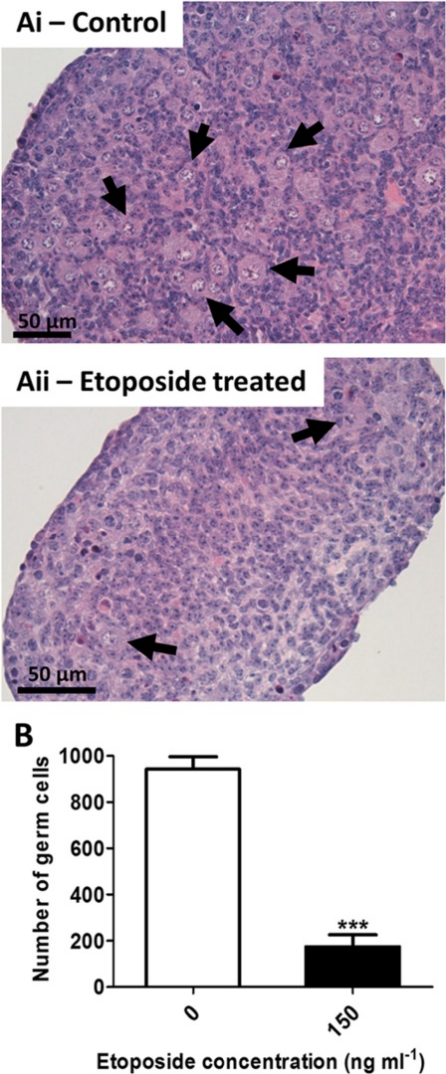
Germ cells show impaired capability of forming follicles in the presence of etoposide. A: Photomicrographs of haemotoxylin and eosin stained E13.5 CD1 ovaries cultured for 6 days either in control medium (*Ai*) or in medium supplemented with the highest dose of etoposide (150 ng ml^−1^; *Aii*). By day 6 of culture, there were significantly fewer germ cells remaining in etoposide-treated ovaries than in controls (**b**). Arrows denote germ cells within the ovary. Scale bars: both 50 μm. Bars denote mean ± SEM, *n* = 5 for controls, *n* = 6 for treatment group. Stars denote significant differences relative to control (***p < 0.0001)

Over the twelve days of culture, oocytes entered meiotic prophase and formed follicles, with some follicles then initiating growth to the primary stage. At the end of culture, histological sections of cultured ovaries were examined (Fig 4Ai-iii), follicles counted and assessed for health. Etoposide had a markedly detrimental effect on total follicle numbers, with a dose-dependent loss of follicles of 72.5 % and 89.7 % at the medium and high doses respectively (Fig. 4Bi, *p* < 0.01 at 100 ng ml ^−1^, *p* < 0.001 at 150 ng ml^−1^; *n* = 6, 2 independent cultures). The observed follicle reduction was due to a loss in PF and transitional follicles within these ovaries (Fig. 5Ai, *p* < 0.01; *n* = 6). The loss was particularly marked for PFs, which constitute over 75 % of the follicles in control ovaries, with numbers reduced to 3.7 % of controls after exposure to 150 ng ml^−1^, compared to 25 % of transitional follicles remaining. The percentage of follicles assessed as unhealthy also increased in a dose dependent manner with increasing etoposide dose, reaching significance at the highest dose of etoposide (Fig. 4Ci, *p* < 0.05; *n* = 6). Again, when each follicle stage was examined, this was seen to be due to a significant increase in the percentage of PF and transitional follicles assessed as unhealthy (Fig. 5Bi, *p* < 0.05; *n* = 6). Ovaries were then examined histologically at day 6 of culture, considered as equivalent to the last day of gestation, a time point at which germ cells are beginning to form follicles (Fig. 6Ai,ii). Ovaries cultured in the presence of the highest dose of etoposide (150 ng ml^−1^) had markedly and significantly fewer germ cells than controls, indicating that the reduced number of follicles observed at the end of culture (Figs. 4Bi and 5Ai) was due to germ cell loss prior to follicle formation (Fig. 6B, *p* < 0.0001, *n* = 5 for controls, *n* = 6 for treatment group).

To investigate the effects of etoposide on folliculogenesis after germ cells are already enclosed in follicles, neonatal mouse ovaries were cultured for six days in control medium or exposed to etoposide (50, 100, 150 or 200 ng ml^−1^) for the duration of culture. Over the six days of culture, some PFs initiated growth to the transitional or primary stage, with a few reaching the secondary stage. In contrast to the fetal ovary culture experiments, when oocytes were exposed to etoposide after follicle formation, no effect was seen on total follicle number (Fig. 4Bii, *p* = 0.092; *n* = 6, 4 independent cultures), or the percentage of unhealthy follicles (Fig. 4Cii, *p* = 0.082), despite exposing neonatal ovaries to a higher concentration of etoposide (200 ng ml^−1^, compared with the highest dose of 150 ng ml^−1^ for fetal ovary cultures). When each follicle stage was examined individually, the only effect seen was at the transitional stage, which accounts for 15 % of follicles in control ovaries. Here, etoposide significantly decreased transitional follicle numbers at 100, 150 and 200 ng ml^−1^ doses (Fig. 5Aii, *p* < 0.05, *p* < 0.01 and *p* < 0.05 respectively; *n* = 6) with a corresponding increase in the percentage of transitional follicles assessed as unhealthy only at the highest dose (Fig. 5Bii, *p* < 0.01; *n* = 6). There was no significant effect on the number or health of PFs (*p* = 0.101, *p* = 0.173 respectively) or primary follicles (*p* = 0.604, *p* = 0.129 respectively).

Overall, results show a marked effect of etoposide on follicle number only when ovaries are exposed prior to follicle formation (fetal ovary culture), with significantly fewer follicles remaining even when exposed to doses as low as 100 ng ml^−1^. In contrast, exposure of ovaries to etoposide only after follicle formation (neonatal ovary culture), has no effect on overall follicle number or health even up to the highest dose of 200 ng ml^−1^, with the only significant effect found specifically on transitional stage follicles.

### Etoposide affects different follicular cell types depending on the time of exposure

**Fig. 7:**
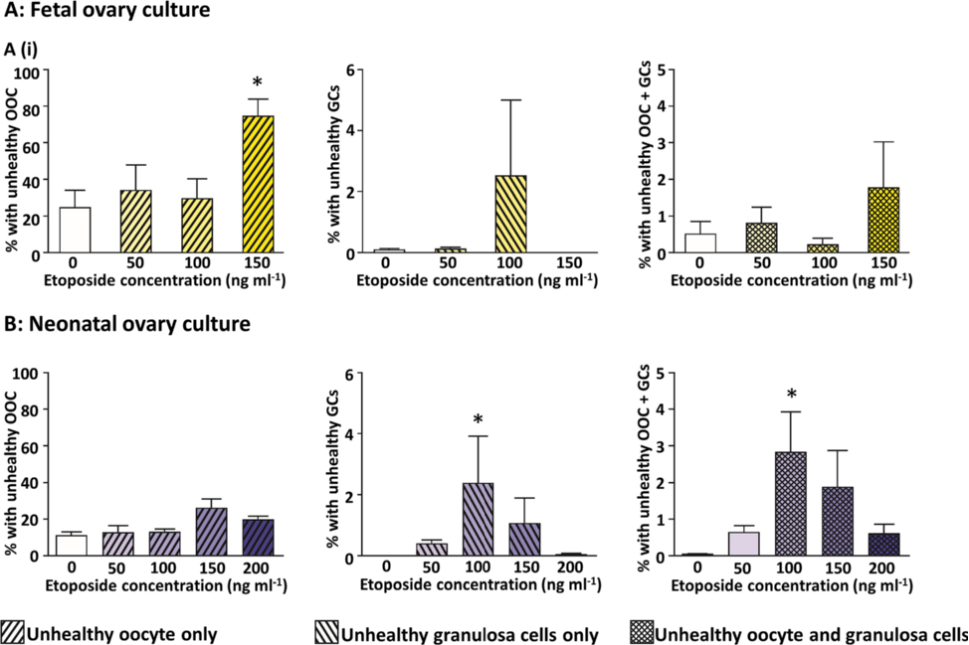
Etoposide primarily targets oocytes prior to follicle formation and granulosa cells after follicle formation. Follicles were categorised as unhealthy due to: unhealthy oocyte (OOC) only (*i*); unhealthy granulosa cells (GCs) only (*ii*); or unhealthy oocyte and granulosa cells (OOC + GCs) (*iii*), in fetal (**a**) or neonatal (**b**) mouse ovaries exposed to etoposide. Bars denote mean ± SEM; *n* = 6 for all groups. Stars denote significant differences relative to control (*p < 0.05)

Figure 4 showed the appearance of unhealthy follicles with unhealthy oocytes in cultured fetal ovaries, exposed to etoposide only prior to follicle formation (Fig. 4Aiii: red arrows), in contrast to the unhealthy follicles with unhealthy granulosa cells in cultured neonatal ovaries, exposed to high concentrations of etoposide only after follicle formation (Fig 4Avi: yellow arrows). In order to examine this in more detail, all histological sections were assessed further, with each unhealthy follicle classified as having: (i) an unhealthy oocyte only; (ii) unhealthy granulosa cells only; or (iii) unhealthy oocyte and granulosa cells. Exposure of fetal ovaries to etoposide resulted in a significant increase in the proportion of follicles with morphologically unhealthy oocytes (Fig. 7Ai, *p* < 0.05), with no effect observed either on the proportion of follicles with unhealthy granulosa cells only or unhealthy oocyte and granulosa cells (Fig. 7Aii,iii, *p* = 0.429 and 0.470 respectively). In contrast, neonatal cultured ovaries exposed to etoposide only after follicle formation, exhibited a significant increase in the proportion of follicles assessed as unhealthy due to unhealthy granulosa cells, or where both the oocyte and granulosa cells were unhealthy (Fig. 7Bii, Biii, *p* < 0.05; *n* = 6), with no significant effect on the proportion of follicles assessed as unhealthy due to an unhealthy oocyte (Fig. 7Bi, *p* = 0.069; *n* = 6).

### Topo IIα is expressed in the female germ cell only prior to follicle formation

**Fig. 8:**
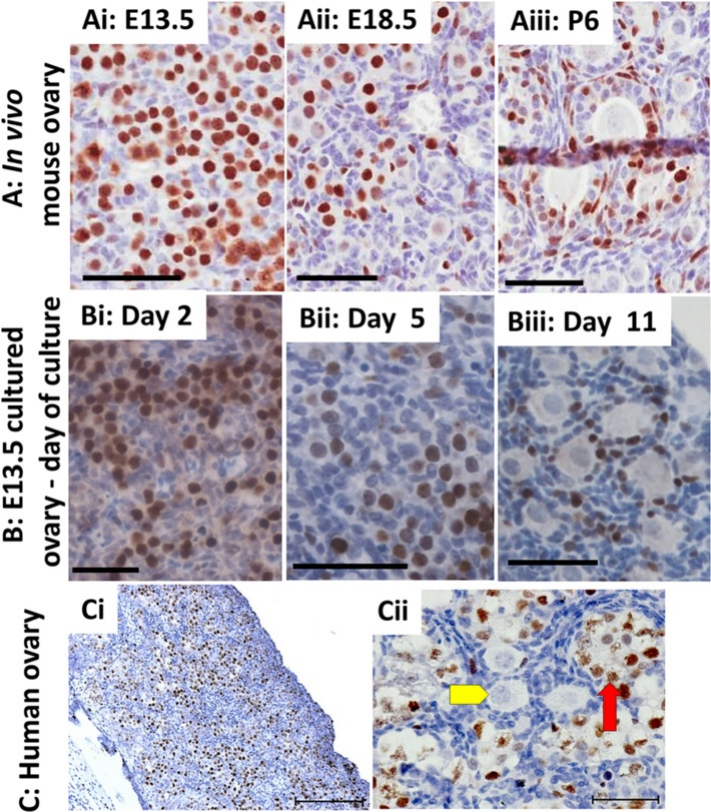
Topo IIα is expressed in the germ cell only prior to follicle formation in the mouse and human ovary. **a**, **b** Topo IIα was localised within the germ cells during pre-natal mouse ovary development (*Ai,ii*), and during the equivalent time in vitro, for the first 5 days of culture (*Bi,ii*). After follicles formed, Topo IIα expression was no longer found within germ cells, instead localised to surrounding somatic cells, both in vivo (*Aiii*) and in vitro (*Biii*). **c** Topo IIα expression was localised to germ cells not yet enclosed in follicles, in ovaries from week 19 human embryos (*Ci, Cii*). *Cii*: red arrow denotes example of Topo IIα expression in germ cell prior to follicle formation, while yellow arrowhead denotes example of absence of Topo IIα expression in germ cell enclosed within ovarian follicle. Scale bars: all 50 μm, except Ci where 200 μm

Lastly, we sought to investigate why etoposide was having more pronounced effects on oocyte rather than granulosa health in fetal ovaries, but less predominantly affecting the somatic granulosa cells rather than the oocyte in neonatal ovaries. Etoposide functions by inhibiting Topo II, preventing it from re-ligating the double-stranded DNA breaks that Topo II introduces [22, 24, 25]. TopoIIβ has been previously reported to be expressed in oocytes at all developmental stages, and is also expressed in granulosa cells of PF and growing follicles [29]. TopoIIα plays a more widespread role in resolving replication-induced topological structures than TopoIIβ [revieved in Refs 24 and 28] and could also be contributing to etoposide sensitivity in the developing ovary. Immunohistochemistry was used to assess Topo IIα expression within the developing mouse ovary, both in vivo from E13.5 through to P6 and in vitro throughout the course of the fetal ovary culture. It was also investigated in a second trimester fetal human ovary, a developmental stage at which the ovary contains female germ cells both prior to and subsequent to follicle formation. In the mouse, both in vivo and in vitro, Topo IIα expression changed from a germ cell-only location prior to follicle formation, to a somatic cell-only location after follicle formation, primarily in granulosa but also expressed in some stromal cells (Fig. 8a, b). Similarly, in the developing human ovary, Topo IIα was expressed in germ cells only prior to follicle formation, although here Topo IIα was no longer expressed after follicle formation (Fig. 8c). The damaging effect of etoposide on ovarian germ cells (Figs. 4, 5 and 6) is, therefore, pronounced only when etoposide exposure is coincident with Topo IIα germ cell-specific expression. Thus the developmental regulation of Topo IIα expression could be one of the factors contributing to the more pronounced effect of etoposide on fetal ovaries, when germ cells express Topo IIα.

## Discussion

The Topo II inhibitor etoposide has been administered to women during pregnancy, with healthy babies born, although there are no data on the side-effects of etoposide on the reproductive system of these children, as none have yet reached puberty. Our results, using in vitro systems to assess the effects of etoposide, show that exposure of pre-follicular ovaries to etoposide results in a near-complete elimination of healthy follicles by the end of culture. This observed lack of follicles is a direct result of etoposide-treated pre-follicular germ cells failing to survive, with only a small proportion capable of forming follicles. Etoposide was used in concentrations considerably lower than those measured in the serum of patients, concentrations of 50–150 ng ml^−1^ being used, compared with 5–60 μg ml^−1^ in patient serum [38]. In contrast, exposure to oocytes once they were enclosed in follicles had no effect on total follicle numbers or health, the only effect seen on transitional follicles, and only when ovaries were exposed to a higher dose of 200 ng ml^−1^. Thus once follicles have formed, the oocytes are less susceptible to etoposide-induced damage. One possible explanation for this change is that Topo IIα was found to be expressed in female germ cells only prior to follicle formation, in both mouse and human. Despite the obvious differences between the mouse ovary cultures described here and the real-life situation of exposure to a human fetus, the results obtained do indicate a cause for concern, especially since etoposide has already been prescribed to pregnant women. Setting aside inter-species differences, it is possible that since etoposide targets mouse germ cells in our culture system, that a similar effect might be observed in a human fetal ovary exposed in vivo. This is especially true if etoposide inhibits Topo IIα in the human ovary, the expression pattern of which is the same in both the human and mouse ovary. Reassuringly, results here showed little evidence of an effect of etoposide on the ability of oocytes to progress through early prophase I of meiosis. Etoposide-treated oocytes were able to reach the diplotene stages of meiosis by the end of culture, although there was an effect of etoposide treatment at Day 2 of culture only, at which point a higher proportion of etoposide-treated oocytes had progressed through leptotene and zygotene to reach the pachytene stage, perhaps indicating that the female germ cells are sensitive to etoposide at mitotic and/or pre-leptotene stages. However, the ability of cultured oocytes in this study to progress through early meiosis without delay does not rule out the possibility that they may have developed DNA damage-induced mutations or chromosomal re-arrangements following etoposide treatment. There may therefore be underlying genetic or chromosomal abnormalities present within these oocytes, which would only become evident much later in oogenesis during meiotic chromosome segregation, or even in a resulting embryo.

Etoposide has previously been shown to directly inhibit the action of Topo II [25–28, 39], and our results here are consistent with this, in particular indicating that the effect of etoposide is most noticeable in cells expressing both Topo IIα and β. When etoposide was administered prior to follicle formation, during the time-frame that both Topo IIα and β are expressed in female germ cells, follicle loss along with a rise in the number of follicles with unhealthy oocytes was observed. In contrast, when etoposide was administered after follicle formation, when Topo IIα is no longer expressed in germ cells, although expression of Topo IIβ continues [29], only subtle germ cell effects were found. Our finding that the oocyte’s sensitivity to etoposide correlates with developmental stages when both Topo IIα and β are expressed, rather than expression of Topo IIβ alone, is consistent with findings in proliferating cell types that Topo IIα provides essential functions that cannot always be efficiently complemented by Topo II β [reviewed in 28]. On the other hand, after follicle formation, when Topo IIα and β are both expressed in granulosa cells, there is a corresponding increase in the proportion of follicles with unhealthy granulosa cells. The sensitivity of fetal oocytes and post-natal granulosa cells to etoposide presumably reflects Topo II activity in these cells, but further work will be required to determine whether the effects of etoposide on oocytes and granulosa cells are caused by DNA double strand breaks generated by etoposide-mediated inhibition of Topo II [25, 26, 28], or by defects in transcription, chromosome structure or chromosome segregation which are all influenced by Topo II activity [24].

In vitro organ and cell culture has become a widely used tool within the field of reproductive toxicology, providing a pragmatic and mechanistic method to study the actions of reproductive toxicants. There are, however, limitations to this method, in that it is difficult to account for any indirect action of a compound that can modulate hormone-signalling pathways. Furthermore, in vitro methods are unable to take into consideration metabolism of the compound, with an effect observed in vivo not necessarily being replicated in vitro. Despite this, in vitro systems have become an invaluable preliminary screening method to discover and investigate potentially harmful compounds on the reproductive system. In vitro systems also have the added advantage that they can reduce the number of animals required for in vivo studies. Work here uses a novel fetal ovary culture system. The method supports female germ cell entry into meiosis, nest breakdown, entry into meiotic arrest, follicle formation and initiation of follicle growth, and allows formation of healthy follicles in ratios comparable to that observed in the neonatal mouse ovary in vivo. Relatively few previous culture systems have been attempted that cover progression into prophase I of meiosis [40–46], and these have not supported subsequent development to the physiological end point of ovarian follicles without the use of invasive techniques [47]. As such, although we have not developed this novel culture system to support follicle growth beyond the primary follicle stage, it could provide an important tool for investigations into early ovary development, including toxicological studies.

## Conclusions

Overall, our results show that early stage female germ cells, prior to follicle formation, are particularly susceptible to levels of etoposide that are low relative to patient serum levels. This effect of etoposide was shown to coincide with a change in Topo IIα expression, with Topo IIα expressed only within female germ cells prior to follicle formation, in the developing ovary of both mouse and human. Our findings indicate the potential for adverse effects on fetal ovarian development when etoposide is used as a chemotherapeutic agent in pregnant mothers during the second or third trimesters, at which point pre-follicular female germ cells are sensitive to the detrimental effects of this compound: effects at this stage of ovary development may well not become apparent until many years later.

## Methods

### Animals

All experiments were approved by the University of Edinburgh’s Local Ethical Review Committee and carried out in accordance with UK Home Office regulations under the ASPA 1986 act. Wild-type CD-1 mice were maintained and bred in an environmentally-controlled room on a 14-h light:10-h dark photoperiod. To obtain fetuses for fetal ovary culture experiments, mouse breeding harems were set up and females checked for the presence of a copulation plug, then designated as E0.5.

### Ovary culture

#### Fetal ovary culture

Pregnant timed-mated females were obtained at E13.5 and culled by cervical dislocation. Genital ridges containing the developing fetal ovaries with mesonephros attached were dissected from female embryos and placed in 1x PBS at 4 °C (day of dissection: Day 0). Ovary-mesonephros complexes were cultured for a total of 12 days on a 2 % agar block (Sigma Aldrich Ltd, Dorset UK) in a 33 mm petri dish, incubated at 37 °C, 5 % CO_2_: see Additional file 1 for further detail. During the first three days of culture (Days 0–3), culture medium contained Dulbecco’s Minimal Essential medium (Life Technologies, Paisley, UK) supplemented with 10 % fetal calf serum (Thermo Fisher, Loughborough UK), 2 mM L-glutamine (Invitrogen), 10 μM β-mercaptoethanol (Life Technologies), 1 % sodium pyruvate (Sigma Aldrich), 1 % penicillin/streptomycin (Invitrogen, Paisley UK) and 1 % amphotericin B (Sigma Aldrich). For the subsequent 9 days of culture (Days 3–12), culture medium was replaced with a simpler culture medium composed of α-MEM medium (Invitrogen) supplemented solely with 3 mg ml^−1^ bovine serum albumin (Sigma Aldrich). Medium was topped up with approximately 200 μl of fresh medium daily, and replaced totally with fresh medium every 72 h.

The effect of etoposide on ovary development was assessed by adding varying doses of etoposide (Sigma Aldrich) to the medium for the first 6 days of culture (Days 0–6). Etoposide was dissolved in DMSO (Sigma Aldrich), therefore DMSO was also added to control medium, with all media containing 0.1 % DMSO. Etoposide was added to produce final concentrations of 50, 100 or 150 ng ml^−1^ (highest concentration of etoposide used was determined from preliminary experiments to find the lowest dose that resulted in the death of the majority of follicles: data not shown). Ovaries were then moved to drug-free culture medium for a further six days (Days 6–12). At the end of culture (Day 12), ovaries were fixed and processed for immunohistochemical or histological analysis. All treatments were *n* = 6, based on initial unpublished work examining ovary development over the time-course of this culture method.

#### Neonatal ovary culture

P0 female mice were culled by decapitation and ovaries dissected out into Leibovitz L-15 dissection medium (Invitrogen) supplemented with 3 mg ml^−1^ bovine serum albumin (Sigma Aldrich); day of dissection: Day 0. Ovaries were cultured for 6 days in a 24-well culture plate (Greiner Bio-one, Stonehouse UK), on Whatman nucleopore polycarbonate membranes (Camlab Ltd, Cambridge UK, 13 mm, 8.0 μm) floating on α-MEM medium (Invitrogen) supplemented with 3 mg ml^−1^ bovine serum albumin, incubated at 37 °C, 5 % CO_2_. Culture medium was supplemented with varying doses of etoposide to produce final concentrations of 50, 100, 150 or 200 ng ml^−1^ for the duration of the culture (Days 0–6). As with the fetal ovary cultures, etoposide was dissolved in DMSO, which was therefore also added to control medium, all media containing 0.1 % DMSO. All treatments were *n* = 6, with previously published work showing this to be sufficient group size [34].

### Histological follicle assessment

At the end of culture, ovaries were placed in 10 % buffered formalin (Sigma Aldrich) for 24 h at room temperature, paraffin wax-embedded, sectioned at 5 μm and stained with haemotoxylin and eosin. Every sixth section was photomicrographed and follicle counts along with assessment of follicle stage and health was carried out, with the observer blind as to treatment. A follicle was included in analysis only where the analysed section contained an oocyte with a visible germinal vesicle. A follicle was considered to be at the PF stage if it contained only flattened pre-granulosa cells, at the transitional stage if it contained both flattened and cuboidal granulosa cells, or at the primary or secondary stage where there was one or two complete layer(s) of cuboidal granulosa cells respectively. Follicle health was recorded onto a separate sheet, with follicles only considered healthy if there was a round oocyte containing a central nucleus and evenly stained cytoplasm, and in the absence of pyknotic granulosa cells. Oocytes were considered unhealthy if they contained a shrunken and pyknotic nucleus or granulosa cells, identified by a dark eosin stain. The Abercrombie correction factor was applied to raw counts to estimate total follicle number per ovary [48].

### Immunohistochemistry

#### Sycp3 immunohistochemistry: meiotic progression in mouse oocytes

Progression through meiotic prophase I was monitored by assessing SC assembly, using antibodies to Sycp3. E13.5 ovaries were cultured in the presence or absence of etoposide (150 ng ml^−1^) for 2, 4 or 6 days. Ovaries were fixed in 10 % buffered formalin, sectioned, dewaxed, rehydrated and antigen retrieval carried out (0.01 M citrate buffer, pH 6). Endogenous peroxidase activity was blocked with 3 % hydrogen peroxide (H_2_O_2;_ Sigma Aldrich) in methanol for 30 mins. Slides were washed (1x phosphate buffered saline [PBS], 0.1 % Triton X-100, Sigma Aldrich) and then incubated for 1 h with 20 % normal goat serum (1x PBS, 5 % bovine serum albumin [BSA]) to block non-specific binding. Sections were incubated overnight at 4 °C with primary mouse anti-Sycp3 antibody (Abcam, 97672), diluted 1:200 in 20 % goat serum (1x PBS, 5 % BSA), washed (1x PBS, 0.1 % Triton X-100) and then incubated for 60 mins with Alexa Fluor 568 secondary anti-mouse antibody (Invitrogen, A21124) diluted 1:200. For visualisation, slides were incubated in DAPI counterstain (Invitrogen, D3571) diluted 1:10000 for 20 mins, washed, mounted using Vectashield (Vector, H-1400) and coverslipped. Photomicrographs (Leica A6000 fluorescent microscope) were obtained for the largest cross section of each cultured ovary. For analysis, counts were made of all oocyte nuclei in leptotene, zygotene, pachytene or diplotene stage of meiotic prophase, with an average of 160 oocytes counted per ovary (*n* = 961 control oocytes, *n* = 994 etoposide oocytes).

#### Localisation of Topo IIα

CD1 mouse ovaries were collected each day from E13.5 embryos through to P6 pups, along with ovaries collected from the fetal ovary culture (Days 1–12). Human ovarian tissue was obtained following elective termination of pregnancy, with informed written consent and approval from the SE Scotland Ethics Committee (reference number 06/S1102/4). Immunohistochemistry was carried out as above with changes as detailed. Antibodies used were: primary – rabbit anti-Topo II α antibody (Abcam, ab52934), diluted 1:100; secondary - biotinylated goat anti rabbit antibody (Dako UK Ltd, Cambridgeshire, UK), diluted 1:200. For visualisation, slides were incubated 30 mins using ABC kit (Vector labs, Peterborough, UK), washed and incubated in DakoCytomation EnVision + DualLink System, Peroxidase (DAB+) solution (Vector labs). Reaction was quenched in H_2_O, slides washed, counterstained for 30 s in haematoxylin, dehydrated and mounted with DPX and glass coverslips.

### Statistical analysis

Statistical analyses were conducted using Graphpad prism (GraphPad Software, Inc., La Jolla, CA, USA). Proportions of germ cells in different stages of meiosis were compared using chi squared analysis. For all other studies involving more than one dose group, data were analysed as follows. Data normality was assessed using Kolmogorov Smirnoff tests. Where data were normally distributed, one way ANOVA was used to detect statistically significant differences across treatments, followed by Bonferroni post-hoc test where ANOVA showed statistical significance. Where data were not normally distributed, Kruskal-Wallis non-parametric test was used, followed by Dunns post-hoc test where Kruskal-Wallis showed statistical significance. Unpaired two-tailed t-tests were used when analyses compared only two groups. Raw data are provided in Additional file 2.

## Abbreviations

E, embryonic day; P, postnatal day; PF, primordial follicle; SC, synaptonemal complex; Topo II, type II Topoisomerase

## Additional files

Additional file 1: Images of embryonic ovaries in culture. (PDF 378 kb) Additional file 2: Full data set for Figures 2-7. (XLSX 35 kb)
