# Supplementary material for: Etoposide damages female germ cells in the developing ovary
Source: BMC Cancer. 2016 Aug 11;16:482. doi: 10.1186/s12885-016-2505-9 (PMC4980800; doi:10.1186/s12885-016-2505-9)
Supplement: Additional file 1: — Images of embryonic ovaries in culture. (PDF 378 kb) [file 12885_2016_2505_MOESM1_ESM.pdf]

Additional file 1: Images of embryonic ovaries in culture

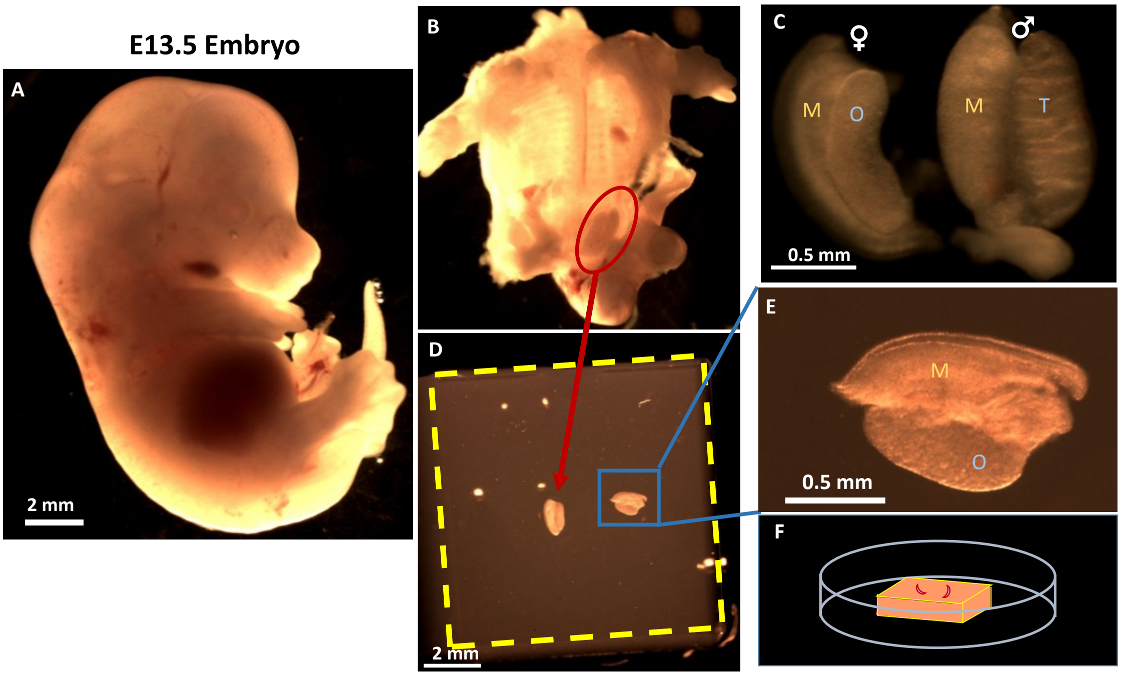

Figure S1. Images of E13.5 mouse ovaries in culture.

E13.5 CD1 mouse pups are collected and placed in a petri dish containing 1xPBS (**A**). A smell ventral incision is made in the embryo, with the heart, bowl and liver removed out of the peritoneal cavity, leaving visible the mesonephros and the genital ridges (**B**, circled in red). At this stage of development, the female gonad (**C**, left) can be distinguished from the male one (**C,** right) by the characteristic transverse sex cords present in the male gonad that will later become the seminiferous tubules, giving it a striated pattern. The ovary is thinner and lacks any visible cords (**C**)**.** The female gonads, along with the mesonephros, are dissected from each side of the body cavity and placed on top of the agar block, with 2-3 ovaries co-cultured on each block, with culture medium at the level of the agar block (**D,** the agar block is outlined by yellow dotted line)**. E:** higher magnification of an ovary in culture on the agar block. **F:** an illustrated figure of the petri dish containing an agar block at approx. 0.5mm height, containing two ovaries (not to scale).

Abbreviations: M= mesonephros, T= testis, O = ovary.
